# Supplementary material for: The ecological consequences and evolution of retron-mediated suicide as a way to protect Escherichia coli from being killed by phage
Source: PLoS One. 2023 May 5;18(5):e0285274. doi: 10.1371/journal.pone.0285274 (PMC10162544; doi:10.1371/journal.pone.0285274)
Supplement: S1 File — (DOCX) [file pone.0285274.s001.docx]

**Supplemental Material - The ecological consequences and evolution of retron-mediated suicide as a way to protect *Escherichia coli* from being killed by phage**

Brandon A. Berryhill, Joshua A. Manuel, Rodrigo Garcia, Bruce R. Levin

**Supplemental Equations**

#

$$\frac{\boldsymbol{dR}}{\boldsymbol{dt}}\boldsymbol{=-}\boldsymbol{\Psi}\left( \boldsymbol{R} \right)\boldsymbol{\cdot}\boldsymbol{e}\boldsymbol{\cdot}\left( \boldsymbol{v}_{\boldsymbol{n}}\boldsymbol{\cdot}\boldsymbol{N+}\boldsymbol{v}_{\boldsymbol{nr}}\boldsymbol{\cdot}\boldsymbol{N}_{\boldsymbol{r}}\boldsymbol{+}\boldsymbol{v}_{\boldsymbol{e}}\boldsymbol{\cdot}\boldsymbol{E+}\boldsymbol{v}_{\boldsymbol{er}}\boldsymbol{\cdot}\boldsymbol{E}_{\boldsymbol{r}} \right)$$

# SEq. (1)

#

$$\frac{\boldsymbol{dN}}{\boldsymbol{dt}}\boldsymbol{=}\boldsymbol{\Psi}\left( \boldsymbol{R} \right)\boldsymbol{\cdot}\left( \boldsymbol{v}_{\boldsymbol{n}}\boldsymbol{\cdot}\boldsymbol{N-}\boldsymbol{\delta}_{\boldsymbol{n}}\boldsymbol{\cdot}\boldsymbol{N}\boldsymbol{\cdot}\boldsymbol{P+}\left( \boldsymbol{\mu}_{\boldsymbol{rn}}\boldsymbol{\cdot}\boldsymbol{N}_{\boldsymbol{r}}\boldsymbol{-}\boldsymbol{\mu}_{\boldsymbol{nr}}\boldsymbol{\cdot}\boldsymbol{N} \right) \right)$$

# SEq. (2)

#

$$\frac{\boldsymbol{d}\boldsymbol{N}_{\boldsymbol{r}}}{\boldsymbol{dt}}\boldsymbol{=}\boldsymbol{\Psi}\left( \boldsymbol{R} \right)\boldsymbol{\cdot}\left( \boldsymbol{v}_{\boldsymbol{nr}}\boldsymbol{\cdot}\boldsymbol{N}_{\boldsymbol{r}}\boldsymbol{-}\left( \boldsymbol{\mu}_{\boldsymbol{rn}}\boldsymbol{\cdot}\boldsymbol{N}_{\boldsymbol{r}}\boldsymbol{-}\boldsymbol{\mu}_{\boldsymbol{nr}}\boldsymbol{\cdot}\boldsymbol{N} \right) \right)$$

# SEq. (3)

#

$$\frac{\boldsymbol{dE}}{\boldsymbol{dt}}\boldsymbol{=}\boldsymbol{\Psi}\left( \boldsymbol{R} \right)\boldsymbol{\cdot}\left( \boldsymbol{v}_{\boldsymbol{e}}\boldsymbol{\cdot}\boldsymbol{E+}\left( \boldsymbol{\mu}_{\boldsymbol{re}}\boldsymbol{\cdot}\boldsymbol{E}_{\boldsymbol{r}}\boldsymbol{-}\boldsymbol{\mu}_{\boldsymbol{er}}\boldsymbol{\cdot}\boldsymbol{E} \right) \right)$$

# SEq. (4)

#

$$\frac{\boldsymbol{d}\boldsymbol{E}_{\boldsymbol{r}}}{\boldsymbol{dt}}\boldsymbol{=}\boldsymbol{\Psi}\left( \boldsymbol{R} \right)\boldsymbol{\cdot}\left( \boldsymbol{v}_{\boldsymbol{er}}\boldsymbol{\cdot}\boldsymbol{E}_{\boldsymbol{r}}\boldsymbol{-}\left( \boldsymbol{\mu}_{\boldsymbol{re}}\boldsymbol{\cdot}\boldsymbol{E}_{\boldsymbol{r}}\boldsymbol{-}\boldsymbol{\mu}_{\boldsymbol{er}}\boldsymbol{\cdot}\boldsymbol{E} \right) \right)$$

# SEq. (5)

#

$$\frac{\boldsymbol{dP}}{\boldsymbol{dt}}\boldsymbol{=}\boldsymbol{\Psi}\left( \boldsymbol{R} \right)\boldsymbol{\cdot}\left( \boldsymbol{\delta}_{\boldsymbol{n}}\boldsymbol{\cdot}\boldsymbol{\beta}_{\boldsymbol{n}}\boldsymbol{\cdot}\boldsymbol{P}\boldsymbol{\cdot}\boldsymbol{N-q}\boldsymbol{\cdot}\boldsymbol{\delta}_{\boldsymbol{e}}\boldsymbol{\cdot}\boldsymbol{P}\boldsymbol{\cdot}\boldsymbol{E+}\left( \boldsymbol{1-q} \right)\boldsymbol{\cdot}\boldsymbol{\delta}_{\boldsymbol{e}}\boldsymbol{\cdot}\boldsymbol{\beta}_{\boldsymbol{e}}\boldsymbol{\cdot}\boldsymbol{P}\boldsymbol{\cdot}\boldsymbol{E} \right)$$

# SEq. (6)

#$\boldsymbol{\Psi}\left( \boldsymbol{R} \right)\boldsymbol{=}\frac{\boldsymbol{R}}{\boldsymbol{R+K}}$

# SEq. (7)

**Figure S1.** Computer simulation results for the effect of the retron efficiency as values of q. Changes in the densities of a retron^+^ bacterial population in the presence (orange) of phage (red) at 24 hours. Shown are 50 simulations with values of q ranging from 0.95 to 1 with a step size of 0.001. The parameters were the same as in Figure 2.

**Table S1.** Parameter values used in the simulations.

**
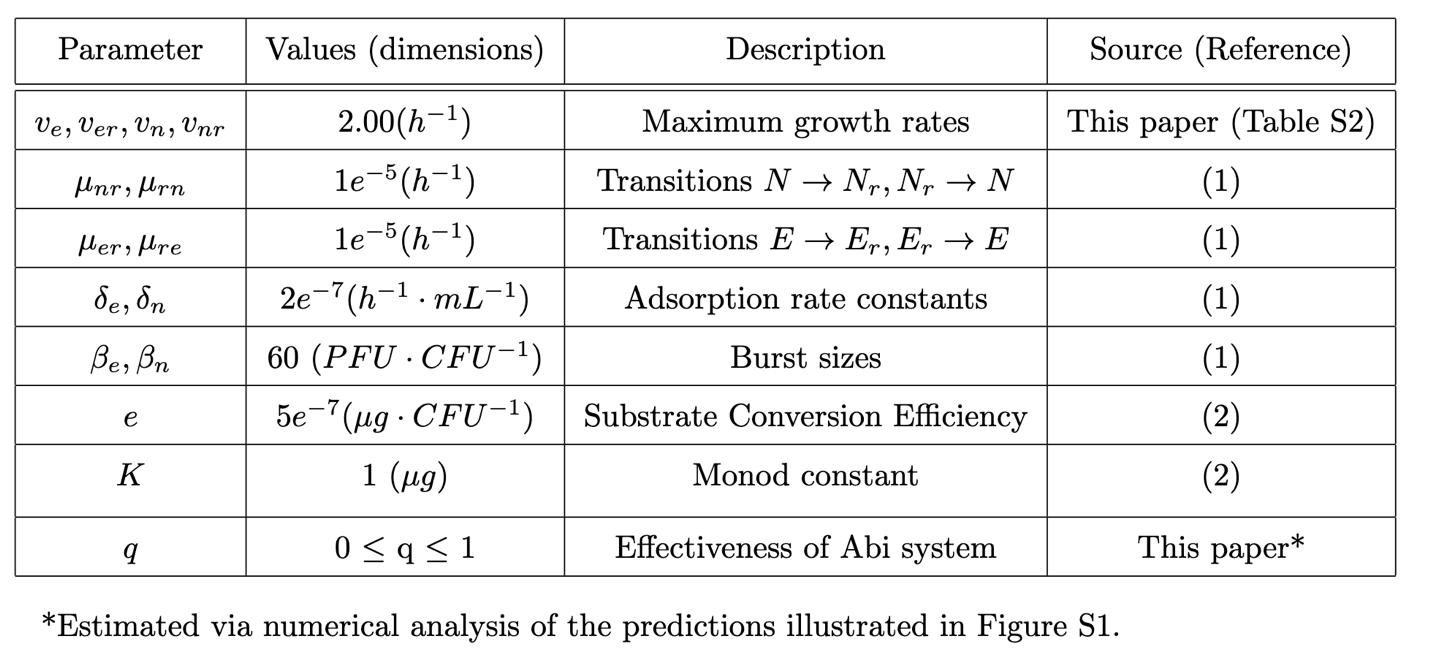
**

1*e*^-5^, 0 (*h*^-1^)

1*e*^-5^, 0 (*h*^-1^)

**Table S2.** Growth rate determination.


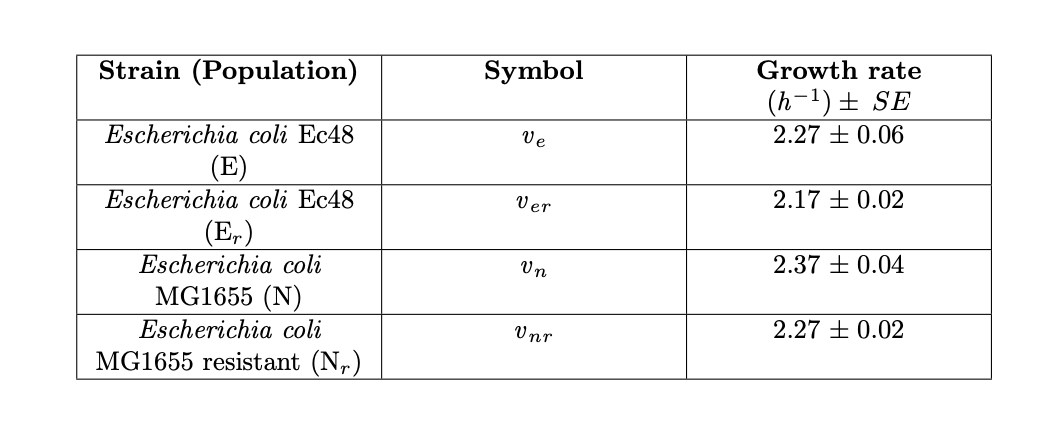


**Table S3.** Cross-streak results at 24 hours.

**References**

1. Chaudhry, W. N., et al. (2018). "Leaky resistance and the conditions for the existence of lytic bacteriophage." PLoS Biol **16**(8): e2005971.
2. Stewart, F. M. and B. R. Levin (1973). "Resource partitioning and the outcome of interspecific competition: a model and some general considerations." American Naturalist **107**: 171-198.
